# The Cost of Expanding Ethiopia’s Salt Iodization Program to Include Multiple Micronutrients

**DOI:** 10.1016/j.cdnut.2025.107508

**Published:** 2025-07-30

**Authors:** Katherine P Adams, Dawd Gashu, Elias Asfaw Zegeye, MG Venkatesh Mannar, Levente L Diosady, N Ananth, E Louise Ander

**Affiliations:** 1 Department of Nutrition, Institute for Global Nutrition, University of California, Davis, CA, United States; 2 Center for Food Science and Nutrition, Addis Ababa University, Addis Ababa, Ethiopia; 3 Economics Department, University of KwaZulu-Natal, Durban, South Africa; 4 Department of Chemical Engineering & Applied Chemistry, University of Toronto, Toronto, Canada; 5 Nutrition Impact Solutions Inc., Toronto, Canada; 6 School of Biosciences, University of Nottingham, Sutton Bonington Campus, Loughborough, Leicestershire, United Kingdom; 7 Inorganic Geochemistry, Centre for Environmental Geochemistry, British Geological Survey, Nottingham, United Kingdom; 8 Africa Centers for Disease Control and Prevention, Health Economics and Financing Division, Addis Ababa, Ethiopia

**Keywords:** large-scale food fortification, multiple fortified salt, cost model, Ethiopia, folic acid, zinc, vitamin B-12

## Abstract

**Background:**

With near universal consumption of salt and technological advances that have made its fortification with multiple micronutrients feasible, salt has great potential for public health impact as a delivery vehicle for not only iodine but also multiple micronutrients. Decisions around modifying existing salt standards to include additional micronutrients should consider not only potential impacts but also stakeholder-specific costs.

**Objectives:**

We aimed to estimate the total and incremental cost of expanding Ethiopia’s salt iodization program to include folic acid (dual fortified salt), folic acid and vitamin B-12 (triple fortified salt), or folic acid, vitamin B-12, and zinc (quadruple fortified salt).

**Methods:**

We developed activity-based cost models to estimate salt fortification costs over a 10-y time horizon (2024–2033). Model assumptions and parameters were primarily based on interviews with Ethiopian stakeholders in industry and government and nongovernmental partners.

**Results:**

The estimated annual average per capita cost of Ethiopia’s existing salt iodization program was ∼$2.1 million (2021 US dollars) or ∼$7/metric ton (MT) of fortified salt (∼$0.02/y). Expanding the program to include folic acid, which could be sprayed onto salt along with the iodine, would increase the annual average per capita cost to ∼$2.5 million, or ∼$8.30/MT (∼$0.02/y). Annually, the costs of triple and quadruple fortified salt programs, which would require encapsulating the additional micronutrients as a solid premix to help ensure stability, were ∼$18 million (∼$59/MT; $0.13 per capita) and $19 million (∼$63/MT; ∼$0.14 per capita), respectively. Premix costs accounted for approximately half of the total cost of the iodized and dual fortified salt programs and ∼90% of triple and quadruple fortified salt.

**Conclusions:**

If Ethiopia considers modifying its existing salt iodization standard to include 1 or more additional micronutrients, there will be many important considerations, including costs and affordability. The cost estimates presented in this study can complement evidence of the potential for multiple fortified salt to reduce micronutrient deficiencies.

## Introduction

Micronutrient deficiencies, sometimes referred to as hidden hunger, remain a significant public health problem globally [[1], [2], [3], [4]]. The consequences of micronutrient deficiencies can be severe, including adverse pregnancy outcomes [low birthweight, preterm birth, and neural tube defects (NTDs), among others], impaired growth and cognitive development, decreased immune function, decreased work capacity, and mortality, all generating significant costs to individuals and societies [1,[5], [6], [7]].

In Ethiopia, the economic cost of nutrient deficiencies and other forms of malnutrition is high, estimated to cost 16.5% of Ethiopia’s annual gross domestic product [8]. An estimated 72% of the Ethiopian population is zinc deficient based on serum zinc [9]. The majority of women (77.9%) have low red blood cell folate concentrations, consistent with increased risk of NTD-affected pregnancies [10]. Although there are no current estimates of the national rate of NTDs in Ethiopia, a systematic review of 15 studies reported a pooled prevalence of 63.3 NTD cases per 10,000 children [11], a figure considerably higher than the worldwide prevalence of 18.6 per 10,000 live births [12]. On the contrary, 8% of women and adolescent girls and 15% of children are deficient in iron and <10% of women and adolescent girls are deficient in vitamin B-12 [13]. These figures are low compared with those of zinc and folate in Ethiopia and, especially for iron deficiency, are low relative to global estimates. In a recent study that brought together biomarker data from 22 low-income and high-income countries, the prevalence of iron deficiency among preschool children was >15% in 17 of the 22 countries and was >8% in 18 of the 20 countries among nonpregnant women of reproductive age (WRA) [4]. Vitamin B-12 deficiency was <10% in 3 of the 9 countries among children and in 9 of the 15 countries among WRA.

Globally, iodine deficiency has been largely addressed through the introduction of universal salt iodization programs. In 1994, the WHO recommended the iodization of salt to help eliminate iodine deficiency and associated health risks [14]. Since then, global recognition of the importance of programs and policies to reduce sodium intake as a strategy to address the growing burden of noncommunicable diseases (NCDs) and NCD-related mortality has sparked debate about the compatibility of universal salt iodization and sodium reduction strategies [15]. However, through coordinated policies, monitoring and surveillance of population iodine and sodium intakes, communication and advocacy, and investments, the WHO promotes universal salt iodization and sodium reduction strategies as compatible and cost-effective strategies to both reduce the global burden of NCDs and control iodine deficiency disorders [16,17]. Today, salt iodization programs have been implemented on either a mandatory or voluntary basis in 150 countries [18], and as of 2020, 88% of the households worldwide consume salt that is fortified with iodine [19].

In the 1990s, Ethiopia introduced a universal salt iodization program. Although the program has been marked by periods of success with high coverage alongside periods of low coverage following times of conflict, currently ∼89% of households consume adequately iodized salt [[20], [21], [22]]. More recently, Ethiopia introduced legislation mandating the fortification of wheat flour with zinc and B vitamins, including folic acid and vitamin B-12 [23]. However, fortifiable wheat flour is consumed by only 30% of the population, mostly concentrated in urban areas [13]. Adherence to iron–folic acid (IFA) supplementation among pregnant women is also low (41.4%) [24], and contrary to the WHO recommendation to avert NTDs [25], a majority of women (71.5%) tend to start taking IFA supplements at a later stage of their pregnancy [26]. Further, given the links between periconceptual folate status and risk of having an NTD-affected pregnancy [27], implementing strategies to improve folate concentrations among women before pregnancy is critical for NTD prevention [28]. Food or condiment fortification with folic acid has been identified as a feasible, effective strategy for improving maternal folate status and preventing NTDs, provided the program is well-designed and implemented, including choosing a food vehicle that is consumed regularly by most of the population and adherence to fortification standards is monitored and enforced [28,29].

The success of salt iodization programs along with its near universal consumption in fairly consistent and self-limiting quantities have spurred interest in salt as a vehicle to safely and effectively deliver other micronutrients [15]. Double fortification of salt with iodine and iron has been introduced in India and reaches millions of consumers, primarily through social safety net programs [[30], [31], [32], [33]]. Technology to add folic acid, vitamin B-12, and/or zinc in addition to iron has also been developed [34] and is being assessed for nutritional impact in India [35,36]. Recent dietary modeling work showed the potential for multiple fortified salt to reduce the prevalence of zinc inadequacy by 6–51 percentage points and folate inadequacy by 2–56 percentage points among the Ethiopian population [37], and a randomized trial to assess the nutritional impact and safety of salt fortified with iodine and folic acid is currently underway in Ethiopia [38]. In terms of consumer acceptability, a recent study in Tanzania found that both double fortified and quadruple fortified salt were equally acceptable to consumers as standard iodized salt [39], and a recent acceptability trial in Ethiopia found that, despite slight color changes (from white to faint yellow), fine and coarse salt sprayed with potassium iodate and folic acid were acceptable to consumers in urban and rural Ethiopia [40]. Despite this growing body of evidence on the potential for salt to deliver multiple micronutrients to populations, including those that are often hard to reach with other micronutrient interventions, not much is known about the cost of multiple fortified salt, especially outside the context of double fortified salt in India.

The Micronutrient Action Policy Support (MAPS) project cocreated (with stakeholders and potential end-users) a web-hosted tool to estimate micronutrient deficiency risks and explore pathways to improve the micronutrient adequacy of diets (https://micronutrient.support/). The cost-effectiveness module of the tool enables users to estimate and compare the cost and cost-effectiveness of alternative micronutrient intervention programs. Using adapted versions of cost models that we developed for use in the MAPS tool, our objectives were to present estimates of the cost of Ethiopia’s salt iodization program, and the total and incremental cost of expanding Ethiopia’s salt fortification program to also include *1*) folic acid (dual fortified salt), *2*) folic acid and vitamin B-12 (triple fortified salt), and *3*) folic acid, vitamin B-12, and zinc (quadruple fortified salt). These estimates will provide policymakers in Ethiopia with important evidence of the potential cost implications to different stakeholder groups of expanding the current salt iodization standard to provide 1 or more additional micronutrients.

## Methods

### Multiple fortification technology

Salt in Ethiopia is currently iodized at industrial-scale salt-refining facilities, with salt typically iodized by spraying the salt with potassium iodate [22]. Fortification of salt with iodine and folic acid can be accomplished by adding folic acid dissolved in sodium carbonate (to maintain alkaline conditions) to the potassium iodate solution and spraying the micronutrients together [34,41,42]. As such, salt refineries in Ethiopia could dual fortify salt with iodine and folic acid without any additional equipment and with minimal changes to their current fortification processes. The addition of vitamin B-12 and/or zinc along with folic acid is more complicated. Testing has shown that vitamin B-12 is unstable when included in a spray solution containing folic acid [34], and iodine degrades quickly when exposed to zinc fortificants in a spray solution [43]. Salt fortification including folic acid and vitamin B-12 and/or zinc is best accomplished when iodine is sprayed and the additional micronutrients are extruded and mixed with the iodized salt [34,43].

The extrusion process involves first mixing the micronutrient premix with semolina flour, vegetable oil, and water and then extruding the dough in a cold forming extruder fitted with a very fine angel-hair pasta die. The extrudate is then cut to match the size of grains of salt then shaped using a spheronizer and dried. The extruded fortified salt-like grains (i.e., extruded premix) then undergo color masking with a spray of a color masking agent (such as titanium dioxide) and finally are encapsulated by coating the particles with hydroxypropyl methylcellulose and soy stearin [43]. With technology transferred from the University of Toronto, JVS Foods Pvt Ltd in Jaipur, India, has the capacity to produce, at large scales, extruded premix for salt fortified with iodine and iron [44,45]. JVS also has the capacity to produce extruded multiple micronutrient premix [35,45]. Given this, we assumed that for triple and quadruple fortified salt, the extruded premix would be produced at the JVS facility, imported into Ethiopia, and distributed to salt refineries. Regardless of where the extruded premix is produced, to support triple or quadruple salt fortification in Ethiopia, refineries would require the installation of blending equipment (including a ribbon or screw blender) to mix the extruded premix with iodized salt to produce multiple fortified salt.

### Cost models

We developed a set of activity-based cost models to estimate the economic cost of the current salt iodization program in Ethiopia and the hypothetical cost of expanding that program to mandate dual (iodine and folic acid), triple (iodine, folic acid, and vitamin B-12), or quadruple (iodine, folic acid, vitamin B-12, and zinc) fortified salt. The cost models, which were developed in Microsoft Excel, were structured based on the set of activities required to plan, execute, and manage each alternative salt fortification program, with each fortification program assumed to mandate the fortification of all salt according to national standards. For each activity, we identified a series of inputs that go into performing the activity (e.g., labor and supplies) and populated each activity in the model with the estimated number of units and associated unit cost of each input needed to undertake the activity.

Costs were estimated over a 10-y time horizon (2024–2033), which allowed for 2 y of planning and initiation activities associated with expanding Ethiopia’s salt iodization program to include additional micronutrients. Costs were defined from a societal perspective, meaning all costs were included regardless of who incurs them. As such, cost estimates accounted for costs that might be paid by the salt industry (refineries), the Ethiopian government, and development partners, as well as costs potentially passed on to salt consumers. Costs were expressed in 2021 United States dollars (USDs). For input costs reported in USD, where necessary we adjusted the value to 2021 USD using the Bureau of Economic Analysis implicit price deflators for gross domestic product [46]. For input costs reported in Ethiopian Birr, we first adjusted to the 2021 value using the local consumer price index [47] and then converted to USD using the average 2021 exchange rate. Given the devaluation of the Ethiopian Birr in 2024 and high annual inflation rates [48], cost estimates would be higher if they were presented in nominal Ethiopian Birr.

### Cost model assumptions and parameter values

The model developed to estimate the cost of Ethiopia’s current salt iodization program comprised strictly recurring costs in each year of the 10-y time horizon. These recurring costs included the cost of potassium iodate, including shipping and local storage and transportation (note that Ethiopia does not tax micronutrient premix, so no taxes or import duties were included). Recurring costs also included industry-related costs to fortify the salt (including labor, power/fuel, annualized equipment costs, and maintenance costs), costs to conduct internal quality assurance and quality control activities at refineries (e.g., internal quantitative iodine tests via titration and WYD spectrophotometer, external laboratory tests), and overhead/administration. Finally, they included government regulatory and monitoring costs (refinery inspections and monitoring, import, commercial, and household monitoring), training/retraining of government personnel, social marketing and advocacy, and overhead/administration.

In addition to the abovementioned recurring costs, each model developed to estimate the cost of expanding the salt fortification program to include multiple micronutrients also included 2 y of expansion costs to account for costs associated with government planning for and revision of the national salt fortification standards, the acquisition of new refinery/fortification facility equipment needed to blend the extruded premix with iodized salt (relevant for triple and quadruple fortified salt), the acquisition of new equipment needed for monitoring, and training for industry personnel involved in salt fortification and training/capacity building for government personnel involved in monitoring of the salt fortification program. We assumed that salt iodization would continue as usual in years 1 and 2 and that salt fortification with multiple micronutrients would begin in year 3 and continue through the end of the modeling time horizon. For the dual fortified salt scenario, activities contributing to recurring costs were very similar to those for iodized salt except the additional cost of folic acid for spraying onto salt alongside potassium iodate as well as some additional monitoring costs to test for folic acid in salt. For the triple and quadruple fortified salt scenarios, recurring costs also accounted for the additional cost of the extruded premix (including shipping and local storage and handling), additional labor, power/fuel, equipment, and equipment maintenance cost, and additional management/overhead. Because we assumed recurring government monitoring activities would be focused on testing for iodine and folic acid, government monitoring costs were the same for dual, triple, and quadruple fortified salt.

For all scenarios, underlying model assumptions and parameter values, including unit costs and quantities, were based on primary data collection, local and international expert input, and secondary data sources. We conducted interviews with salt refineries in Ethiopia to understand the activities required to iodize salt and conduct quality assurance and quality control and to generate estimates of the associated personnel and supply costs. We also conducted interviews with government personnel involved in monitoring of the salt iodization program to accurately characterize current monitoring and enforcement activities and to generate estimates of the cost of undertaking those activities. Some model assumptions and parameter values were informed by secondary data, including published standards for salt iodization in Ethiopia, United Nations World Population Prospects for national population projections over the 10-y time horizon [49], and published and gray literature. Equipment costs were annualized using a 3% discount rate and assuming a useful life of 10 y.

For dual, triple, and quadruple fortified salt, we modeled 1 different potential fortification concentrations (Table 1) [37,50,51]. The first set of fortification concentrations (scenario 1) were based on current salt consumption patterns (7.4 g/d among WRA) estimated from urinary sodium excretion in the National STEPS (or STEPwise approach to surveillance) survey [50] and assuming 90% of salt intake is from discretionary intake and manufactured food items [37]. The second set of fortification concentrations (scenario 2) were based on a hypothetical scenario in which discretionary salt consumption in Ethiopia decreased to ∼5 g/d, in line with WHO recommendations to limit sodium intake to <2000 mg/d [52]. Except for iodine, in both scenarios, the fortification concentrations were developed based on modeling of dietary data in Addis Ababa and the Somali Region (regions known for lowest and highest micronutrient deficiency, respectively, compared with other regions), with the fortification concentrations for each salt consumption concentration selected to maximize micronutrient intake and also ensure risk of high intake remained < 5% [37]. For scenario 1, we used Ethiopia’s current salt iodization standard (30 mg/kg), whereas for scenario 2, we assumed salt would be iodized to 39 mg/kg, in line with WHO guidelines when salt consumption is ∼5 g/d among adults [53]. Folic acid, vitamin B-12, and iodine in fortified refined salt are stable for 6 mo of storage and high temperatures (45 °C) [34]. As such, we did not include overages in the premix formulations. Note that cost estimates did not account for costs associated with sodium reduction campaigns or the cost of revising fortification standards in response to reduced salt consumption concentrations. Given the low prevalence of iron deficiency in Ethiopia (estimated to be 8% among WRA and adolescent girls and 13% among children 6–59 mo) [13], we did not model the inclusion of iron. The prevalence of vitamin B-12 deficiency is also low (<10% among WRA and adolescent girls) [13], but we included vitamin B-12 because it plays an important role in folate metabolism and could therefore improve the effectiveness of fortifying salt with folic acid [54].

**TABLE 1:** Salt fortification cost modeling scenarios.

	Scenario 1—current salt consumption^1^				Scenario 2—reduced salt consumption^2^			
	Iodized salt	Dual fortified salt	Triple fortified salt	Quadruple fortified salt	Iodized salt	Dual fortified salt	Triple fortified salt	Quadruple fortified salt
Average per capita discretionary salt consumption (g/d)	6.7	6.7	6.7	6.7	5	5	5	5
Iodine (mg/kg)	30	30	30	30	39	39	39	39
Folic acid (mg/kg)	0	17.2	17.2	17.2	0	23.4	23.4	23.4
Vitamin B-12 (mg/kg)	0	0	0.39	0.39	0	0	0.39	0.39
Zinc (mg/kg)	0	0	0	600	0	0	0	800

1 Average discretionary salt consumption estimates for scenario 1 based on average salt intake among women of reproductive age of 7.4 g/d in Ethiopia estimated from urinary sodium excretion in the National STEPS survey [50] and assuming 90% of salt intake is from discretionary intake and manufactured food items [37].

2 Average discretionary salt consumption for scenario 2 based on recommendations from WHO to reduce sodium intake to <2000 mg/d, which is equivalent to 5 g of salt/d, for adults [51].

For sprayed micronutrients (potassium iodate for each type of fortified salt and folic acid for dual fortified salt), we estimated premix costs using a premix cost calculator that was developed to enable estimation of the cost of micronutrient premix for fortified salt and other food vehicles with the flexibility to adjust fortification concentrations, micronutrient fortificants, and fortificant prices. Fortificant prices in the premix cost calculator were informed by estimates provided by an international large-scale food fortification program expert in November 2021. Because the triple and quadruple salt fortification scenarios involved extruded and encapsulated micronutrients that we assumed would be produced in India and imported into Ethiopia, the cost estimates of the extruded premixes were based on estimates provided by JVS Foods Pvt Limited as informed by the company’s experience producing premixes for efficacy trials in India in 2021 and validated by market cost estimates obtained from Indiamart (www.indiamart.com). As JVS Foods Pvt Limited is currently the only private premix manufacturer with the technology and capacity to produce extruded fortified premix for multiple fortified salt, these estimates represent the best available source of cost data. However, in recognition of uncertainty around these cost estimates, we conducted sensitivity analyses around these and other uncertain parameters (described further). Recent estimates of industry compliance with Ethiopia’s salt fortification standard suggest that most (89%) of the salt is fortified with iodine [55]. As such, we modeled costs assuming 89% of discretionary salt and salt used in manufactured food items is fortified to the national standard.

### Sensitivity analyses

Because multiple fortified salt is a hypothetical micronutrient intervention in Ethiopia, some of our cost model parameter values were based on cost data from other contexts as well as informed assumptions about how a multiple salt fortification program in Ethiopia would be designed and implemented. As such, our study was not designed to allow for statistical testing of differences in costs across scenarios. However, to assess the influence of uncertain parameter values on the total and incremental cost of expanding Ethiopia’s salt iodization program to include additional micronutrients, for the current salt consumption patterns scenario (scenario 1), we conducted several sets of sensitivity analyses around particularly uncertain parameters.

First, because micronutrient premix is often the most expensive component of a large-scale food fortification program and because, apart from salt fortified with iodine and iron, multiple fortified salt has not been produced for use at scale and our cost estimates are based on cost data from 1one company, we varied the price of the micronutrient premix up and down by 30% (including the cost of potassium iodate). We also recognize uncertainty around the additional costs salt refineries would face to fortify salt with multiple micronutrients, so we also varied up and down by 30% the total cost of salt refinery fortification costs, which includes labor, power/fuel, annualized equipment costs, and equipment maintenance costs. There is also uncertainty in how the government of Ethiopia would monitor a multiple fortified salt program, so we assessed the impact of this uncertainty by varying total government monitoring costs (including refinery monitoring, salt import monitoring, and household monitoring) up and down by 30%. Finally, we estimated best-case and worst-case scenarios by simultaneously decreasing the price/cost of each of these uncertain parameters by 30% (the best-case scenario) and simultaneously increasing their price/cost by 30% (the worst-case scenario).

### Ethical review

The co-design of the MAPS tool was approved by the University of Nottingham Research Ethics Committee (study reference 1920-087-BIO). The University of California, Davis Institutional Review Board deemed the cost study exempt from human subjects oversight.

## Results

### Primary results

We estimated that, over the 10-y time horizon of 2024–2033, the annual average per capita cost of Ethiopia’s current salt iodization program was ∼$2.1 //million (2021 USD), equating to ∼$7 per metric ton (MT) of fortified salt and ∼$0.02/y (Table 2 [49], scenario 1). Expanding the current program to include folic acid was estimated to increase the annual average cost by ∼$415,000 to ∼$2.5 million and to increase the average cost per MT of fortified salt to ∼$8.30 (note that the cost per capita rounds to ∼$0.02 in both cases, although it is slightly higher for dual fortified salt than that for iodized salt). For triple fortified salt, which involves mixing iodized salt with extruded premix that includes folic acid and vitamin B-12 fortificants, we estimated that the annual average cost would increase to ∼$18 million. Compared with the current iodization program, triple fortified salt was estimated to increase the cost of fortification by ∼$52/MT of salt to ∼$59/MT and increase the annual average per capita cost to $0.13 or ∼$0.11 higher than the cost per capita of Ethiopia’s current salt iodization program. Finally, the estimated annual average cost of quadruple fortified salt was slightly over $19 million or ∼$63 per MT and ∼$0.14 per capita per year.

**TABLE 2:** Estimated total and incremental cost of salt fortification in Ethiopia.

	Iodized salt ($)	Dual fortified salt ($)	Triple fortified salt ($)	Quadruple fortified salt ($)
Scenario 1—current salt consumption				
Total cost^1^				
Annual average total cost	2,115,000	2,531,000	18,009,000	19,014,000
Annual average cost per capita^2^	0.02	0.02	0.13	0.14
Annual average premix cost^3^ per MT of fortified salt	2.7	3.7	43.1	45.7
Annual average total cost per MT of fortified salt	7.0	8.3	59.3	62.6
Incremental cost^4^				
Annual average total cost	—	416,000	15,894,000	16,899,000
Annual average cost per capita^1^	—	0.003	0.11	0.12
Annual average premix cost^2^ per MT of fortified salt	—	1.0	40	43
Annual average total cost per MT of fortified salt	—	1.4	52	56
Scenario 2—reduced salt consumption				
Total cost^1^				
Annual average total cost	1,987,000	2,410,000	17,474,000	18,318,000
Annual average cost per capita^2^	0.01	0.02	0.12	0.13
Annual average premix cost^3^ per MT of fortified salt	3.5	4.9	55.9	58.9
Annual average total cost per MT of fortified salt	8.7	10.6	76.6	80.3
Incremental cost^4^				
Annual average total cost	—	423,000	15,487,000	16,331,000
Annual average cost per capita^1^	—	0.003	0.11	0.12
Annual average premix cost^2^ per MT of fortified salt	—	1.4	52	55
Annual average total cost per MT of fortified salt	—	1.9	68	72

Abbreviations: MT, metric ton.

1 Costs modeled over 10-y time horizon (2024–2033) and reported in undiscounted 2021 US dollars. Total costs are rounded to the nearest thousand and costs per MT are rounded to the nearest tenth.

2 Ethiopia population estimates based on the 2019 World Population Prospects total population estimates, 2024–2033 [49].

3 Premix costs include the cost of micronutrient fortificants, and in the case of triple and quadruple fortified salt, the cost of additional ingredients and the production of extruded fortified salt-like grains.

4 Incremental costs calculated as the difference in cost between iodized salt and the alternative multiple fortified salt scenario.

**FIGURE 1:**
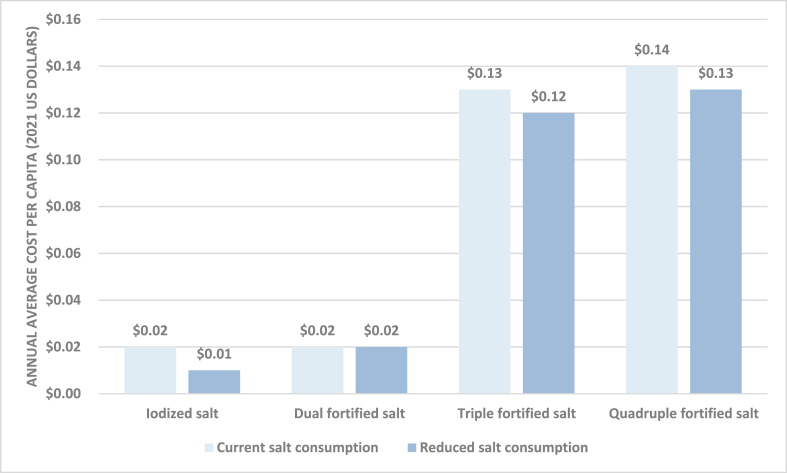
Estimated annual average per capita cost of salt fortification in Ethiopia based on current salt consumption patterns and reduced salt consumption (to 5 g/d).

Based on the hypothetical scenario in which average salt consumption in Ethiopia fell to WHO recommended concentrations of 5 g/d among adults (with modeled fortification concentrations correspondingly increased), the estimated annual average costs of iodized, dual, triple, and quadruple fortified salt would be similar to annual average cost estimates based on current salt consumption patterns (Table 2, scenario 2). However, given that a smaller quantity of fortified salt would be required to meet demand, the average price per MT of fortified salt would be higher. Specifically, the estimated cost per MT of fortified salt was ∼$8.70 for iodized salt, ∼$10.60 for dual fortified salt (iodine and folic acid), ∼$76.60 for triple fortified salt (iodine, folic acid, and vitamin B-12), and ∼$80/MT for quadruple fortified salt (iodine, folic acid, vitamin B-12, and zinc). Although the average price per MT of fortified salt is higher in this scenario than the current salt consumption scenario, the total quantity of fortified salt in the food system is lower in this scenario, resulting in lower total annual average cost estimates and, correspondingly, lower estimates of annual average per capita costs, which are $0.01, $0.02, $0.12, and $0.13, respectively. Per capita cost estimates for both scenarios are summarized in Figure 1.

Activity-specific cost estimates and proportional contributions of each activity to total annual average costs based on current salt consumption patterns (scenario 1) are reported in Table 3. Recurring premix costs for iodized and dual fortified salt made up approximately half of the total intervention program costs (47% and 55%, respectively). This was followed by *1*) recurring industry-related fortification costs, including labor, power/fuel, annualized equipment costs, and equipment maintenance costs, representing 26% of the total salt iodization program costs and 22% of the total cost of double fortified salt, and *2*) recurring refinery management, administration, and overhead costs (16% and 14%, respectively). For the triple and quadruple fortified salt programs, recurring premix costs accounted for >90% of the total annual average cost. Note that the micronutrient fortificants contributed 24% and 54% to the total cost of the extruded, encapsulated premix for triple and quadruple fortified salt, respectively, whereas other ingredients and the manufacturing process accounted for the remainder of the premix costs. In all cases, other start-up and recurring costs made up a small proportion (6% or less) of the total cost of the programs. In terms of the potential distribution of costs across stakeholder groups, refinery-related costs (excluding premix) accounted for ∼49% of the annual average cost of the iodized salt program and ∼41% of the total cost of the dual fortified salt program. Given that premix costs are significantly higher for triple and quadruple fortified salt, these refinery-related costs represented ∼8% of the total cost of these fortification programs. For the existing salt iodization program as well as each hypothetical program, average annual government-related costs were <5% of the total annual average cost.

**TABLE 3:** Activity-specific cost and share of total cost estimates of salt fortification in Ethiopia based on current salt consumption (scenario 1).

	Iodized salt		Dual fortified salt		Triple fortified salt		Quadruple fortified salt	
	Average annual cost (2021 USD)	Percentage of total cost^1^	Average annual cost (2021 USD)	Percentage of total cost^1^	Average annual cost (2021 USD)	Percentage of total cost^1^	Average annual cost (2021 USD)	Percentage of total cost^1^
Start-up/scale-up costs								
Relabeling, annualized cost	0	0	1000	0	1000	0	1000	0
Training for salt refinery personnel, annualized cost	0	0	1000	0	1000	0	1000	0
Government planning (reformulation of standards, adoption/reformulation of M&E plan, etc), annualized cost	0	0	2000	0	3000	0	3000	0
Training/capacity building for government M&E personnel, annualized cost	0	0	2000	0	2000	0	2000	0
Recurring costs								
Premix, including shipping and taxes	1,002,000	47	1,388,000	55	16,432,000	91	17,437,000	92
Salt refinery fortification costs, including labor, power/fuel, annualized equipment costs, and maintenance costs	546,000	26	546,000	22	835,000	5	835,000	4
Salt refinery fortification QA/QC activities	125,000	6	125,000	5	125,000	1	125,000	1
Salt refinery internal training/retraining	22,000	1	22,000	1	22,000	0	22,000	0
Salt refinery management, administration, and overhead related to fortification	347,000	16	347,000	14	491,000	3	491,000	3
Government inspections and monitoring of salt refineries	8000	0	13,000	1	13,000	0	13,000	0
Government monitoring of imported salt	2000	0	3000	0	3000	0	3000	0
Government monitoring of salt at markets and other retail outlets	2000	0	12,000	0	12,000	0	12,000	0
Government household monitoring	14,000	1	14,000	1	14,000	0	14,000	0
Social marketing/advocacy	9000	0	9000	0	9000	0	9000	0
Capacity building/training for food control agency personnel/monitors/laboratory technicians	14,000	1	14,000	1	14,000	0	14,000	0
Government management, administration, and overhead	24,000	1	32,000	1	32,000	0	32,000	0
Total costs								
Annual average premix cost^2^	1,002,000	47	1,388,000	55	16,432,000	91	17,437,000	92
Refinery-related annual average total cost	1,040,000	49	1,042,000	41	1,474,000	8	1,474,000	8
Government-related annual average total cost	73,000	3	101,000	4	103,000	1	103,000	1
Total annual average cost	2,115,000	100	2,531,000	100	18,009,000	100	19,014,000	100

Costs modeled over 10-y time horizon (2024–2033) and reported in undiscounted 2021 USD rounded to the nearest thousand.

Abbreviations: M&E, monitoring and evaluation; QA/QC, quality assurance/quality control; USD, US dollar.

1 Percentage of total cost is rounded to the nearest whole number. When an annual average cost is <0.5% of the total cost, the percentage of total cost is rounded down to 0%.

2 Premix costs include the cost of micronutrient fortificants, and in the case of triple and quadruple fortified salt, the cost of additional ingredients and the production of extruded fortified salt-like grains.

The breakdown of activity-specific costs, their contributions to total costs, and the possible distribution of costs across stakeholder groups based on the reduced salt consumption scenario are presented in Table 4. For each possible salt fortification program, these estimates were very similar to the current salt consumption scenario, with micronutrient premix representing ∼50% of the total annual average cost of the iodized and dual salt fortification programs and >90% for triple and quadruple fortified salt.

**TABLE 4:** Activity-specific cost and share of total cost estimates of salt fortification in Ethiopia based on reduced salt consumption (scenario 2).

	Iodized salt		Dual fortified salt		Triple fortified salt		Quadruple fortified salt	
	Average annual cost (2021 USD)	Percentage of total cost^1^	Average annual cost (2021 USD)	Percentage of total cost^1^	Average annual cost (2021 USD)	Percentage of total cost^1^	Average annual cost (2021 USD)	Percentage of total cost^1^
Start-up/scale-up costs								
Relabeling, annualized cost	0	0	1000	0	1000	0	1000	0
Training for salt refinery personnel, annualized cost	0	0	1000	0	1000	0	1000	0
Government planning (reformulation of standards, adoption/reformulation of M&E plan, etc), annualized cost	0	0	2000	0	3000	0	3000	0
Training/capacity building for government M&E personnel, annualized cost	0	0	2000	0	2000	0	2000	0
Recurring costs								
Premix, including shipping and taxes	978,000	49	1,372,000	57	16,002,000	92	16,845,000	92
Salt refinery fortification costs, including labor, power/fuel, annualized equipment costs, and maintenance costs	477,000	24	477,000	20	765,000	4	765,000	4
Salt refinery fortification QA/QC activities	125,000	6	125,000	5	125,000	1	125,000	1
Salt refinery internal training/retraining	22,000	1	22,000	1	22,000	0	22,000	0
Salt refinery management, administration, and overhead related to fortification	312,000	16	312,000	13	456,000	3	456,000	2
Government inspections and monitoring of salt refineries	8000	0	13,000	1	13,000	0	13,000	0
Government monitoring of imported salt	2000	0	3000	0	3000	0	3000	0
Government monitoring of salt at markets and other retail outlets	2000	0	12,000	0	12,000	0	12,000	0
Government household monitoring	14,000	1	14,000	1	14,000	0	14,000	0
Social marketing/advocacy	9000	0	9000	0	9000	0	9000	0
Capacity building/training for food control agency personnel/monitors/laboratory technicians	14,000	1	14,000	1	14,000	0	14,000	0
Government management, administration, and overhead	24,000	1	32,000	1	32,000	0	32,000	0
Total costs								
Annual average premix cost^2^	978,000	49	1,372,000	57	16,002,000	92	16,845,000	92
Refinery-related annual average total cost	936,000	47	937,000	39	1,370,000	8	1,370,000	7
Government-related annual average total cost	73,000	4	101,000	4	103,000	1	103,000	1
Total annual average cost	1,987,000	100	2,410,000	100	17,474,000	100	18,318,000	100

Costs modeled over 10-y time horizon (2024–2033) and reported in undiscounted 2021 USD rounded to the nearest thousand.

Abbreviations: M&E, monitoring and evaluation; QA/QC, quality assurance/quality control; USD, US dollar.

1 Percentage of total cost is rounded to the nearest whole number. When an annual average cost is <0.5% of the total cost, the percentage of total cost is rounded down to 0%.

2 Premix costs include the cost of micronutrient fortificants, and in the case of triple and quadruple fortified salt, the cost of additional ingredients and the production of extruded fortified salt-like grains.

### Sensitivity analysis

**FIGURE 2:**
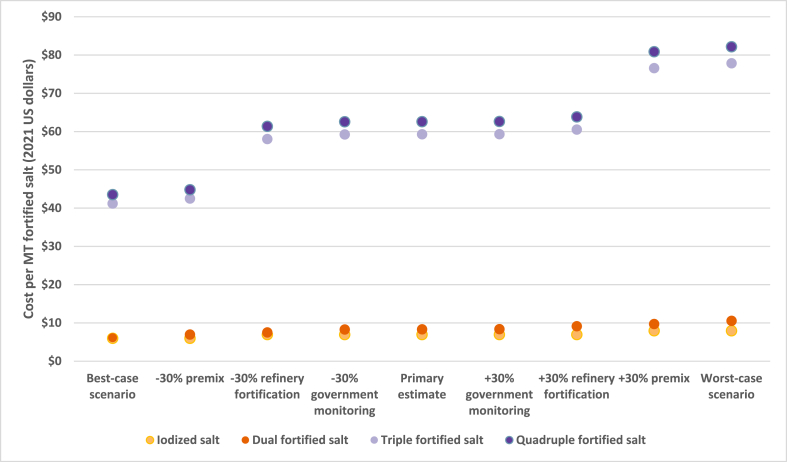
Sensitivity analysis—estimated annual average cost per metric ton (MT) of fortified salt based on current salt consumption patterns (scenario 1) with variation in key cost model inputs.

Variation in the estimated annual average cost per MT of fortified salt across several sources of uncertainty are presented in Figure 2. For each type of fortified salt, varying the price of the micronutrient premix (and the extruded premix in the case of triple and quadruple fortified salt) up and down by 30% had the largest impact on the annual average cost per MT of fortified salt, potentially increasing or decreasing the annual average cost of iodized salt by ∼$1.00/MT, the cost of double fortified salt by ±∼$1.40/MT, and the cost of triple and quadruple salt by ±∼$16–$18/MT. Thirty percent variation in refinery fortification-related costs increased or decreased the cost per MT by ∼$1 for dual, triple, and quadruple fortified salt, whereas 30% variation in government monitoring costs had a small impact (∼$0.05) on the estimated annual average cost per MT. With all sources of cost variation considered simultaneously, the worst-case estimated annual average cost was $10.55/MT for dual fortified salt, $77.85/MT for triple fortified salt, and $82.15/MT for quadruple fortified salt. At the other extreme, the best-case estimates of the annual average cost per MT were $6.11, $41.22, and $43.53 for dual, triple, and quadruple fortified salt, respectively.

## Discussion

Universal salt iodization has played a fundamental role in the global reduction of iodine deficiency disorders, with substantial health and economic benefits [56,57]. Expanding salt iodization programs to fortify salt with multiple micronutrients has the potential to build on this success and improve the adequacy of multiple micronutrients in diets. However, decisions around expanding salt iodization programs should be evidence based. Beyond salt fortified with iodine and iron, the body of evidence on the potential impacts of salt fortified with other micronutrients is growing. An upcoming RCT in the Oromia Region of Ethiopia will provide evidence on the potential impact of salt fortified with iodine and folic acid on the folate status of WRA [38,58]. In Punjab, India, an RCT will provide evidence on the impact of quintuply fortified salt (iodine, iron, folic acid, vitamin B-12, and zinc) on the biomarker status of WRA and preschool-age children [36]. Finally, recent modeling work using dietary recall data showed that multiple-fortified salt could lead to substantial reductions in the prevalence of inadequate zinc and folate intakes in both urban and rural Ethiopia [37]. In this article, based on detailed activity-based cost models, we provide complementary evidence on the potential cost of using salt as a food vehicle to enhance the micronutrient adequacy of diets in Ethiopia beyond iodine.

We found that the estimated annual average per capita costs of Ethiopia’s current mandatory salt iodization program and hypothetical mandatory dual (iodine and folic acid), triple (iodine, folic acid, and vitamin B-12), and quadruple (iodine, folic acid, vitamin B-12, and zinc) salt fortification programs were ∼$0.02, ∼$0.02, $0.13, and ∼$0.14, respectively. More specifically, we found that, from a societal costing perspective and based on current salt consumption patterns, a mandatory dual fortified salt program (iodine and folic acid), which could rely on existing salt iodization technology, would increase the annual average cost of the salt fortification program from ∼$7/MT of fortified salt to ∼$8.30/MT, corresponding to an annual average increase in the cost per capita of <$0.01. The preferred technology for triple (iodine, folic acid, and vitamin B-12) or quadruple (iodine, folic acid, vitamin B-12, and zinc) fortified salt requires extruding the micronutrient fortificants into a salt-like grain that is then encapsulated and mixed with iodized salt [34,43]. We estimated that this extrusion and encapsulation process, in addition to the added cost of the additional micronutrient fortificants, additional refinery fortification-related costs, and additional government monitoring and evaluation costs would increase the total annual average cost of the program substantially, from just over ∼$2 million per year to ∼$18 and $19 million annually, translating to $0.11 and $0.12 higher estimated annual average costs per capita. These additional costs would not only deliver 1 or 2 additional micronutrients but would also likely improve the effectiveness of fortifying salt with folic acid because of the role of vitamin B-12 in folate metabolism [54]. Under a hypothetical scenario in which salt consumption in Ethiopia dropped to an average of 5 g/d among adults, necessitating not only higher fortification concentrations to help meet requirements but also a smaller total quantity of salt in the food system to fortify, the estimated total and incremental costs were similar to those based on current salt consumption patterns. Although not directly comparable due to differences in the included micronutrients and their dosages, these annual average per capita costs are substantially lower than the cost of an 180-d supply of IFA tablets (∼$2.00) or multiple micronutrient supplements (∼$3.42), excluding programmatic costs [59].

The price of iodized salt in Addis Ababa in October 2024 was ∼$0.27/kg for a relatively inexpensive salt packaged in a plastic bag (although the market price varies depending on type of salt, packaging, etc). Relative to this, the incremental cost estimates represent a ∼0.5% increase in the price for dual fortified salt, a ∼19% increase for triple fortified salt, and a ∼21% increase in the price of quadruple fortified salt. Although it is unlikely that all of these incremental costs would be passed on to salt consumers, even if just premix costs were passed on to consumers, this would still represent a sizeable increase in the price of salt in the case of triple and quadruple fortified salt. At the same time, it is important to acknowledge the potential additional health benefits that may be conferred to the population if salt also delivered vitamin B-12 (to both aid in folate metabolism and improve dietary vitamin B-12 adequacy) and/or zinc to deficient populations in Ethiopia. Issues such as affordability and the tradeoff between higher salt prices and public health benefits would need to be considered by decision-makers in Ethiopia. Moreover, although the incremental cost of triple and quadruple fortified salt is low in absolute terms at $0.11–$0.12 per capita per year, the cost relative to iodized and dual fortified salt may be a barrier to adoption in Ethiopia and other low-income and middle-income countries. Future research in this space to identify alternative technologies for multiple fortified salt at a lower cost could help reduce this potential barrier.

The cost estimates presented in this study should be interpreted with several limitations in mind. First, because a multiple fortified salt program in Ethiopia is hypothetical, some of our cost inputs and assumptions about how the program might operate were based on experiences in other contexts and on informed assumptions. We conducted sensitivity analyses to assess the impact of some of this uncertainty, but actual costs incurred in practice may still vary from our estimates. In addition, our cost estimates reflected current industry compliance with the national salt iodization standard. If compliance with a revised standard that required fortifying salt with multiple micronutrients was lower (or higher) than current compliance, this would impact the cost of the program. Related, studies assessing the feasibility of multiple fortified salt have been conducted with dried refined salt [34,[41], [42], [43]]. Although most of Ethiopia’s salt supply for human consumption is based on production processes that produce high-quality, refined salt, some proportion (< 30%) is produced using practices that results in lower-quality salt [22]. As such, the technical feasibility of fortification of lower-quality salt with multiple micronutrients would need to be considered. Another limitation is that our cost estimates for triple and quadruple fortified salt are based on the assumption that the extruded premix would be produced in India and imported into Ethiopia. The quantities of fortified salt to meet the demands of just the Ethiopian market are currently too small to justify a local premix facility, but that could change if a regional market for encapsulated micronutrient fortification developed. Finally, because spraying salt with folic acid has been shown to turn the salt yellow, consumer acceptability of dual fortified salt may require education and advocacy efforts [41]. In India, organoleptic changes to salt as a result of the inclusion of iron have negatively impacted consumer acceptance and uptake, thus curtailing demand for double fortified salt in the open market [32]. Although recent evidence from Ethiopia on the acceptability of dual and triple fortified salt suggests consumer acceptability will not be a significant barrier in that context [40], this may still be an important consideration, particularly if organoleptic changes are more pronounced with, for example, the fortification of lower-quality salt.

Study strengths include the development of detailed, 10-y activity-based cost models, the modeling of several different multiple fortified salt alternatives, and modeling costs based on not only current salt consumption patterns but also a scenario in which salt consumption in Ethiopia decreased to a concentration aligned with the WHO concentrations recommended for sodium reduction. Sodium reduction efforts need not conflict with salt fortification programs [53]. The modeling results based on discretionary salt consumption of ∼5 g/d demonstrate how reductions in salt consumption in response to, for example, sodium reduction campaigns, can be integrated into salt fortification programs by adjusting fortification concentrations to ensure dietary requirements are still met while also enabling understanding the potential cost implications of adjusting fortification standards.

Salt, with its nearly universal consumption across all population groups and recent technological advances that have made its fortification with multiple micronutrients feasible and reliable (i.e., with micronutrient stability and retention) [34], has great potential for public health impact as a delivery vehicle for multiple micronutrients. If Ethiopia considers modifying its existing salt iodization standard to include 1 or more additional micronutrients, there will be many important considerations, including which micronutrients to include and at what fortification concentrations, consumer acceptability of the chosen formulation, how the program would be regulated and enforced, feasibility to adhere to revised standards, particularly for smaller-scale salt refineries, and so on. Another important consideration will be how the cost of the salt fortification program would likely change, which stakeholder groups would be called upon to pay those higher costs, and whether those higher costs would be affordable to each stakeholder group.

This article provides evidence on the potential incremental costs of expanding Ethiopia’s current salt iodization program to include folic acid, folic acid and vitamin B-12, or folic acid, vitamin B-12, and zinc. This evidence can complement evidence on the potential impact of multiple fortified salt. To further build this evidence base, next steps could include estimating the cost-effectiveness and/or cost benefit of multiple fortified salt in Ethiopia.

## Author contributions

The authors’ responsibilities were as follows – KPA, DG, ELA: designed the study; KPA, DG, EAZ, VM, NA: collected the data; LLD: advised on the appropriate multiple fortified salt technology for each scenario; KPA: developed the cost models, wrote the first draft of the manuscript, and has responsibility for the final content; and all authors: contributed to the data interpretation and revisions of the manuscript and read and approved the final manuscript.

## Data availability

The Excel cost model to estimate the cost of salt iodization is publicly and freely available without restriction at https://doi.org/10.5281/zenodo.14564050. Excel cost models to estimate the cost of dual, triple, and quadruple fortified salt will be made available upon request from the corresponding author.

## Funding

This work was supported, in whole or in part, by the Gates Foundation [INV-002855]. The conclusions and opinions expressed in this work are those of the author(s) alone and shall not be attributed to the Foundation. Under the grant conditions of the Foundation, a Creative Commons Attribution 4.0 License has already been assigned to the Author Accepted Manuscript version that might arise from this submission.

## Conflict of interest

KPA, DG, EAZ, and ELA report financial support was provided by Gates Foundation. The other authors report no conflicts of interest.
